# When will ‘open science’ become simply ‘science’?

**DOI:** 10.1186/s13059-015-0669-2

**Published:** 2015-05-19

**Authors:** Mick Watson

**Affiliations:** The Roslin Institute, Royal (Dick) School of Veterinary Studies, University of Edinburgh, Easter Bush, Edinburgh, EH25 9RG UK

## Abstract

Open science describes the practice of carrying out scientific research in a completely transparent manner, and making the results of that research available to everyone. Isn’t that just ‘science’?

## Comment

Open science is the practice of making everything in the discovery process fully and openly available, creating transparency and driving further discovery by allowing others to build on existing work. When I read such definitions, I think ‘but isn’t that just science?’ Sadly not. In his review of Michael Neilsen’s book *Reinventing Discovery* [1], Timo Hannay describes academic science as ‘self-serving’ and ‘uncooperative’, ‘replete with examples of secrecy and resistance to change’ and describes the natural state of researchers as ‘one of extreme possessiveness’ [2]. And who can argue? The majority of publications are behind a paywall, raw data are hidden, methods ill-described, software unreleased and reviews anonymous. Open science is often described as a ‘movement’, bringing to mind images of revolution, a few plucky visionaries fighting against an unfair ruler; but revolution against what? Who is the unfair ruler?

At what point did we allow science to become closed? How did we allow this to happen?

At present, open science is seen as an optional extra, on the fringes of everyday research: open access to articles is offered at additional cost; including raw data in publications isn’t mandatory; anonymous peer review is the default. Imagine the opposite. Imagine having to pay to make your work closed; imagine having to state and then justify why your raw data should remain secret. In other words, imagine if open science was considered normal, and closed science considered weird. Wouldn’t the world be a better place?

Of course, in some cases, privacy and anonymity are justified. However, we should never kill good ideas because of fringe cases. I call this the ‘mobile phone paradox’. The mobile phone is an incredible, world-changing invention that allows two people, anywhere in the world, to communicate with one another. Yet they do not always work, because in some areas there is no signal. Should we have not invented mobile phones because in some cases they will not work? Of course this is ridiculous. The same is true of open science - it will not always work, but it is still the right thing to do.

There are six commonly accepted pillars of open science: open data, open access, open methodology, open source, open peer review and open education.

### Open data

Open data is the process of releasing both raw and processed data from your experiments, enabling others to analyse it without restriction. That data should be released is obvious; but which data? In my opinion, all raw data generated in the pursuit of your experiment should be released (especially the data you discarded), and at least enough to regenerate completely the analysis you yourself performed. As important as the data are the metadata; releasing raw data with poor metadata is just another way of obfuscating the scientific process.

We should consider the data to be the main publication, and the paper a secondary, less important part; the data will outlive the paper, as others re-analyse within the context of new scientific discoveries. Imagine if the human genome project had only released the ‘interesting parts’ of the genome? So many scientific discoveries would have been delayed.

Alongside the scientific argument is the moral argument; as Hannay alludes to, it is no longer acceptable for scientists to hold on to data until they have extracted every last possible publication from it. The data do not belong to the scientist, they belong to the funder (quite often the taxpayer). Datasets should be freely available to those who funded them. Scientists who hoard data, far from pushing back the boundaries of human knowledge, instead act as barriers to discovery.

Of course, we should always be careful to ensure appropriate consent is given, and that data cannot result in the harm of any given individual or group. However, it is ironic that many of us are careless about personal data every day, yet demand that scientific data are held up to a higher standard.

### Open access

Open access is the model under which papers are available for anyone to read without having to pay, and that license allows secondary use such as text-mining. Others have spoken about this at great length, but some points are worth re-iterating: it is immoral to expect those who funded the research (taxpayers) to pay to access the results of that research; it is illogical that researchers (who work for the journals for free) have to pay; or that institutions who employ those authors have to pay. Nothing about the current closed-access publication model makes sense. Who should pay? The funder, of course; and when there is no funder, or there are no funds, then there are preprint servers (such as arXiv and bioRxiv) and institutional repositories.

I have no problem with publishers making money from the scientific process. However, I believe that in order to do so, they should add value. Many will say that they add value; and some do; but many more do not. Typesetting and PDF generation are not ‘adding value’. A good example of ‘adding value’ are the ‘living figures’ introduced by *F1000Research* [3], figures within papers that update in real-time as more data become available. Rather tellingly *F1000Research* is an open-access publisher.

### Open methodology

An open methodology is simply one which has been described in sufficient detail to allow other researchers to repeat the work and apply it elsewhere. Isn’t that simply ‘the methods section’? Of course, there are times when researchers may have access to unique resources - a cell line, or specific computer hardware - which means that others cannot repeat what they did. That doesn’t matter. One of the major reasons we publish is so that others can learn from what we have done, and revealing how you carried out an experiment is at the heart of any publication.

### Open source

Open source generally refers to open and free access to the blueprint of a product; applied to software, it refers to the source code. There are hundreds of different open-source software licenses, and the arguments for and against are detailed and nuanced. However, I refer you to ‘Open methodology’ above; if you use software as part of the scientific method, then the source code should be available to read (preferably via a website such as GitHub or SourceForge), the software should compile and run and there should be a description of the core algorithms. The software you develop is part of the methods section, and it is the easiest part to share. One can distribute software throughout the world at the push of a button; the same cannot be said of a laboratory. Software should be (and in fact is) driving the open-science movement.

### Open peer review

I have written extensively about this [4, 5], as have others [6]. The point of open peer review isn’t removing anonymity, though that’s part of it. Open peer review is about transforming the peer review process; it is about making peer review a collaborative process between authors and reviewers; it is about constructive criticism, but with the goal of helping the authors to get published. More than all of that, it’s about doing the right thing. The *British Medical Journal* gathered convincing evidence that open review did no damage to the quality of peer reviews [7]; yet still they insisted that they introduced open peer review for ‘ethical reasons’, believing that removing anonymity would help bring an end to the worst abuses of peer review, and transform the entire process from one of judgement to one of open, scientific discourse [7]. When reading those words, doesn’t it make you wonder why peer review was ever anything else?

### Open education

Open education refers to the open and free availability of educational resources. This does not mean that you cannot charge for education - no one can make the tutor work for free - but the resources that are used to educate can be made freely available. Why would you do that? So that others can use and improve them, and so that standards can be set and reached. In my own field, bioinformatics, this is being driven by movements such as GOBLET [8, 9] and Software/Data Carpentry [10]. More widely, massively open online courses (MOOCs) are increasingly popular. Open education brings science and education to everyone, regardless of social class, and that can only be a good thing.

## Concluding remarks

Open science isn’t a movement, it’s just (good) science. It’s also the future. Science, and particularly scientific publishing, is at a turning point. It reminds me of retail in the 1990s, just as the internet was beginning to take off. Many huge, successful retailers took one look at the internet and thought ‘That’ll never catch on’. Five years later they were closing stores and winding-up their business as the more innovative and agile internet companies replaced them. Open science is the future, and it will replace closed science. I encourage you to embrace it.
